# Implication of Admission Eosinophil Count and Prognosis of Coronavirus Disease 2019 (COVID‐19) in Elderly Patients With COPD: A Territory‐Wide Cohort Study

**DOI:** 10.1111/crj.70070

**Published:** 2025-03-27

**Authors:** Wang Chun Kwok, Yat Fung Shea, James Chung Man Ho, David Chi Leung Lam, Terence Chi Chun Tam, Anthony Raymond Tam, Mary Sau Man Ip, Ivan Fan Ngai Hung

**Affiliations:** ^1^ Department of Medicine The University of Hong Kong, Queen Mary Hospital Pokfulam Hong Kong China

**Keywords:** chronic obstructive pulmonary disease, COVID‐19, eosinophilic phenotype, SARS‐CoV‐2

## Abstract

**Objectives:**

This study aims to investigate the association between elderly patients with COPD with different blood eosinophil on admission and those without COPD and the prognosis of COVID‐19.

**Method:**

A territory‐wide retrospective study was conducted to investigate the association between elderly COPD patients with different blood eosinophil on admission and the prognosis of COVID‐19. Elderly patients admitted to public hospitals and community treatment facility in Hong Kong for COVID‐19 from January 23, 2020, to September 31, 2021, were included in the study. Severe diseases were defined as those who develop respiratory complications, systemic complications and death.

**Results:**

Among the 1925 patients included, 133 had COPD. Forty had admission blood eosinophil count ≥ 150 cells/μL, and 93 had blood eosinophil count < 150 cells/μL. Patients with COPD and admission blood eosinophil count ≥ 150 cells/μL, but not those with admission blood eosinophil count < 150 cells/μL, had severe COVID‐19 with the development of respiratory and systemic complications. They were more likely to develop respiratory failure (OR = 5.235, 95% CI = 2.088–13.122, *p* < 0.001) and require invasive mechanical ventilation (OR = 2.433, 95% CI = 1.022–5.791, *p* = 0.045) and intensive care unit admission (OR = 2.214, 95% CI = 1.004–4.881, *p* = 0.049).

**Discussion:**

Our study suggested that the blood eosinophil count on admission could have significant prognostic implications among elderly patients with COPD. Patients with COPD and admission blood eosinophil count ≥ 150 cells/μL, but not those with admission blood eosinophil count < 150 cells/μL, have significantly increased risks of developing respiratory and systemic complications from COVID‐19, when compared with non‐COPD patients.

## Graphical abstract

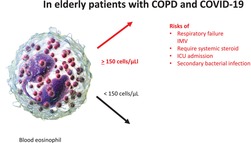

## 1 Introduction

Advance of age is reported to be a risk factor for coronavirus disease 2019 (COVID‐19), which increases the hospitalization rate and mortality [1, 2]. Underlying comorbidities are also associated with severe disease [2, 3]. Among the comorbidities, chronic obstructive pulmonary disease (COPD) was reported to be associated with severe disease, in terms of hospitalization and mortality [4], though this was challenged by other studies.

In recent years, phenotyping of COPD based on eosinophil count has emerged as a way to categorize the disease, which has implication on the treatment regime. Baseline eosinophil level at stable state is one of the used tools to categorize the COPD phenotype [5]. There were also studies using the blood eosinophil count at hospitalization to categorize the COPD phenotype [6, 7]. The role of blood eosinophil count at hospitalization among COPD patients remains controversial, with some suggested that higher blood eosinophil count at admission for a COPD exacerbation was associated with increased COPD readmission rates in patients with infrequent COPD hospitalizations [6], but some suggested eosinophil value below 144 cells/μL on admission or < 2% was associated with longer hospital length of stay and some proposed that higher blood eosinophils were associated with better outcomes in hospitalized COPD patients [7]. Various cutoff for eosinophilic phenotype of COPD was used and 150 cells/μL was one of the most commonly used ones [8, 9, 10, 11, 12].

In view of the possibility of blood eosinophil count at hospitalization among COPD patients may have prognostic implications, there were studies conducted to assess if it could serve as a biomarker to guide the treatment. There were two multicenter randomized controlled noninferiority to suggest that blood eosinophil–guided treatment with systemic steroid to be noninferior compared with standard of care with potential benefits in reducing the duration of systemic corticosteroid exposure and may allow clinicians to safely reduce systemic glucocorticoid use [13, 14]. Recently, the Acute exacerbations treated with BenRAlizumab trial (ABRA) trial suggested the benefits of using benralizumab among treating acute eosinophilic asthma and COPD exacerbations, defined as blood eosinophil count ≥ 300 cells/μL [15], with lower risks of treatment failure compared with steroid treatment alone. The implication of admission blood eosinophil count is one of the areas that worth further investigation to provide personalized therapy among COPD patients.

There are few studies on the association between eosinophil levels on admission among patients with chronic respiratory disease who are hospitalized for COVID‐19. One of the studies suggested that eosinopenia with eosinophil counts less than 20 cells/μL on admission conferred a higher risk of severe disease requiring intensive care but not mortality [16]. But this study included patients with asthma, COPD and obstructive sleep apnea (OSA), in which eosinophil level is not a well‐established biomarker in OSA. The study also did not separate patients from asthma and COPD, who have different clinical characteristics. Another small‐scale study that included 59 patients with COPD and asthma also showed similar findings [17]. But this study also did not separate COPD from asthma patients and severe disease were defined by physiological parameters instead of clinical outcome. An American study suggested that lack of eosinophil recovery defined as reaching ≥ 50 eosinophils/μL may suggest the risks of progression to severe COVID [18]. A study suggested that the association between eosinophil level and COVID‐19 outcomes depends on the use of inhaled corticosteroid. They proposed that blood eosinophil level above 150 cells/μL was associated with improved COVID‐19 outcomes in inhaled corticosteroid‐treated patients [19]. A study conducted in Croatia suggested the possibility of eosinophil count at hospital admission might have a potential prognostic role for all‐cause mortality at 30 days of follow‐up; however, the authors could not demonstrate this among patients' preexisting obstructive lung diseases [20]. Whether eosinophil count alone can predict the prognosis was not examined in this study as this study.

Apart from antiviral, adjunctive treatment including systemic corticosteroid with dexamethasone [21, 22, 23], JAK inhibitor baricitinib [24, 25] and IL‐6 receptor blocker tocilizumab [26, 27] were also suggested to be effective in severe COVID‐19. It is important to identify the patients who are at risks of deterioration by simple biomarkers that may allow early initiation of adjunctive therapies. Combination antiviral was also studied in high‐risk patient subgroups as well [28, 29, 30].

In this current study, we investigated the association between elderly patients with COPD with different blood eosinophil on admission and those without COPD, and the prognosis of COVID‐19, from a territory‐wide cohort.

## 2 Methods

A territory‐wide retrospective study was conducted to investigate the association between elderly COPD patients with different blood eosinophil on admission and the prognosis of COVID‐19. Patients admitted to public hospitals in Hong Kong for COVID‐19 from the January 23, 2020, to September 30, 2021, were included. According to public health policy in Hong Kong, all patients with COVID‐19 regardless of disease severity need to be hospitalized in public hospital or community treatment facility, which are managed by the Hospital Authority under isolation order from the government. Patients with moderate to severe disease are managed in the isolation ward of acute hospital, but mild and asymptomatic cases are managed in Hong Kong Infection Control Centre. Home‐based management is not adopted in Hong Kong. Patients are only allowed to be discharged from hospital when repeated respiratory specimens were negative for SAR‐CoV2 and the isolation order is removed. Patients were identified from Clinical Data Analysis and Reporting System (CDARS) of Hospital Authority. Cases with COVID‐19 were identified by International Classification of Diseases, Ninth Revision code of 519.8. CDARS is an electronic healthcare database managed by the Hospital Authority which covers 90% of healthcare services of Hong Kong, as well as managing all patients with COVID‐19 in Hong Kong. The study was approved by the Institutional Review Board of the University of Hong Kong and Hospital Authority Hong Kong West Cluster (UW 22‐089).

The clinical data including demographics, date of hospitalization, length of stay, development of clinical outcomes and laboratory results were retrieved from CDARS. The first blood eosinophil count at the time of hospitalization was retrieved from CDARS, which was used to define subjects into different subgroups, using the cutoff value of blood eosinophil count was 150 cells/μL. Patients with high eosinophil count at the time of hospitalization was defined as admission blood eosinophil count ≥ 150 cells/μL. But those with admission blood eosinophil count < 150 cells/μL were defined as having low eosinophil count.

Inclusion criteria including age of 65 years old or above with laboratory (RT‐PCR) confirmed COVID‐19. Exclusion criteria include patients with asthma.

The primary outcome is the development of respiratory failure, which is defined as the need of supplementary oxygen, noninvasive, and invasive mechanical ventilation. The secondary outcomes include the need of invasive mechanical ventilation, need of invasive mechanical ventilation for more than 95 h, systemic steroid treatment for COVID‐19, intensive care unit admission, development of ARDS, shock, development of acute kidney injury, in‐hospital mortality, 30‐day mortality, and secondary bacterial and viral infections. Multivariate logistic regression was done to adjust for potential confounders including Charlson Comorbidity Index (CCI), serum albumin level, and lymphocyte count. CCI is a well‐validated method to predict of long‐term prognosis and survival [31, 32, 33]. CCI was also found to have role in risk stratifications of hospitalized COVID‐19 patients [34]. Serum albumin level and lymphocyte count are surrogate markers for nutritional status in elderly patients [35].

### 2.1 Statistical Analysis

The demographic and clinical data were described in actual frequency or mean ± SD. Baseline demographic and clinical data were compared between the patients without COPD and those with COPD and admission blood eosinophil count of different levels by one‐way ANOVA. To identify whether COPD with different blood eosinophil count on admission is associated with severe disease, univariate logistic regression analyses were performed. Multiple logistic regression modeling was used to take into account of potential confounders including age and comorbidities that are known poor prognostic factors of COVID‐19. Length of stay between the three groups was compared by log‐linear regression. The statistical significance was determined at the level of *p* = 0.05. All the statistical analyses will be done using the 26th version of SPSS statistical package.

## 3 Results

### 3.1 Patients' Characteristics

From January 23 2020, to September 30, 2021, 11 292 adult patients with COVID‐19 were admitted to public hospitals in Hong Kong. Among these patients, 1925 of them fulfilled the inclusion criteria, being age above 65 and without history of asthma. Among the 1925 patients included in the analysis, there were 979 (50.9%) male patients, with mean age of 73.9. There were 133 patients with COPD in the cohort, 93 had blood eosinophil count on admission < 150 cells/μL, and 40 had blood eosinophil count ≥ 150 cells/μL. There were 11 patients who had hospitalized COPD exacerbation in the past 1 year before admission for COVID‐19, with 1 patient having 1 hospitalized COPD exacerbation, 3 having 2 hospitalized COPD exacerbation, and 7 had ≥ 3 hospitalized COPD exacerbation in the past 1 year. There were 16 (12.0%) patients with Group A COPD, 82 (61.7%) with Group B COPD and 35 (26.3%) with Group E COPD with no statistically significant difference among the two groups with different admission blood eosinophil count. Among the patients with COPD, 35 (26.3%) of them were prescribed on inhaled corticosteroid (ICS) before admission and are not statistically significant between the groups with different blood eosinophil count. The baseline demographics of the patients were listed in Table 1.

**TABLE 1:** Baseline demographic and clinical characteristics of included patients.

	Patients without COPD (*n* = 1792)	Patients with COPD and admission blood eosinophil count < 150 cells/μL (*n* = 93)	Patients with COPD and admission blood eosinophil count ≥ 150 cells/μL (*n* = 40)	*p*
Age, mean ± SD (range)	73.0 ± 7.3 (65–97)	78.3 ± 11.1 (65–96)	81.6 ± 10.0 (66–95)	< 0.001
Sex				< 0.001
Male	894 (49.9%)	54 (58.1%)	31 (77.5%)	
Female	898 (50.1%)	39 (41.9%)	9 (22.5%)	
Hypertension	1079 (60.2%)	59 (63.4%)	29 (72.5%)	0.247
Diabetes mellitus	575 (32.1%)	29 (31.2%)	16 (40.0%)	0.557
Hyperlipidemia	763 (42.6%)	39 (41.9%)	20 (50.0%)	0.636
Ischemic heart disease	422 (23.5%)	32 (34.4%)	18 (45.0%)	< 0.001
Inhaled corticosteroid use	—	23 (24.7%)	12 (30.0%)	0.527
COPD group				0.772
Group A	—	12 (12.9%)	4 (10.0%)	
Group B	—	58 (62.4%)	24 (60.0%)	
Group E	—	23 (24.7%)	12 (30.0%)	
eGFR, mean ± SD (range)	72.2 ± 15.3 (3–124)	69.8 ± 18.5 (25–100)	65.6 ± 25.4 (26–103)	0.0136
Blood eosinophil count on admission (cells/μL), median, [25th–75th percentile]	21.5 [0–100]	10 [0–100]	300 [200–400]	< 0.001
Serum albumin level (g/L) mean ± SD (range)	38.2 ± 5.2 (13–58)	34.8 ± 5.7 (20–47)	36.2 ± 4.5 (13–58)	< 0.001
Blood lymphocyte count (cells/μL), mean ± SD (range)	142 ± 68.9 (0–4)	120 ± 72.2 (0–5)	147 ± 64.7 (0–3)	0.085
Charlson Comorbidity index median [25th–75th percentile]	3 (3–4)	5 (4–6)	5 (5–7)	< 0.001
Respiratory failure	714 (39.8%)	62 (67.7%)	31 (77.5%)	< 0.001
Invasive mechanical ventilation	155 (8.6%)	23 (24.7%)	10 (25.0%)	< 0.001
Invasive mechanical ventilation of more than 95 h	54 (3.0%)	7 (7.5%)	4 (10.0%)	0.386
Require systemic steroid treatment	666 (37.2%)	56 (60.2%)	29 (72.5%)	< 0.001
Intensive care unit admission	223 (12.4%)	21 (22.6%)	13 (32.5%)	< 0.001
COVID pneumonia	296 (16.5%)	41 (44.1%)	22 (55.0%)	< 0.001
ARDS	119 (6.6%)	10 (10.8%)	3 (7.5%)	0.306
Shock	138 (7.7%)	18 (19.4%)	9 (22.5%)	< 0.001
Acute kidney injury	33 (1.8%)	2 (2.2%)	2 (5.0%)	0.350
Secondary bacterial infection	984 (54.9%)	73 (78.5%)	36 (90.0%)	< 0.001
Secondary viral infection	31 (1.7%)	2 (2.2%)	2 (5.0%)	0.301
Inpatient mortality	105 (5.9%)	17 (18.3%)	9 (22.5%)	< 0.001
30‐day mortality	75 (4.2%)	9 (9.7%)	6 (15.0%)	< 0.001
Median length of stay [25th–75th percentile]	14 [9–21]	19 [12.25–40.75]	23 [14–24]	< 0.001

### 3.2 Disease Severity and Complications for Patients With or Without COPD

On univariate logistic regression, patients with COPD were significantly more likely to develop respiratory failure with odds ratio (OR) of 3.369 (95% CI = 2.476–3.298, *p* < 0.001); require invasive mechanical ventilation (OR = 3.485, 95% CI = 2.275–5.339, *p* < 0.001), the need for invasive mechanical ventilation of more than 95 h (OR = 3.156, 95% CI = 1.553–6.415, *p* < 0.001), and intensive care unit admission (OR = 3.478, 95% CI = 2.361–5.125, *p* < 0.001); have COVID pneumonia (OR = 4.456, 95% CI = 3.163–6.532, *p* < 0.001); require systemic corticosteroid treatment (OR = 2.994, 95% CI = 2.075–4.320, *p* < 0.001); develop shock (OR = 3.053, 95% CI = 1.933–4.821, *p* < 0.001) and secondary bacterial infection (OR = 3.729, 95% CI = 2.374–5.858, *p* < 0.001); and have inpatient mortality (OR = 3.941, 95% CI = 2.458–6.318, *p* < 0.001) and 30‐day mortality (OR = 2.910, 95% CI = 1.621–5.223, *p* < 0.001).

In multivariate logistic regression, patients with COPD were significantly more likely to develop respiratory failure (OR = 2.625, 95% CI = 1.560–4.417, *p* < 0.001); require invasive mechanical ventilation (OR = 2.196, 95% CI = 1.186–4.069, *p* = 0.012), invasive mechanical ventilation of more than 95 h (OR = 4.374, 95% CI = 1.485–12.881, *p* = 0.007), and intensive care unit admission (OR = 1.842, 95% CI = 1.054–3.218, *p* = 0.032); develop COVID pneumonia (OR = 3.054, 95% CI = 1.787–5.219, *p* < 0.001); require systemic corticosteroid treatment (OR = 2.565, 95% CI = 1.550–4.244, *p* < 0.001); and develop secondary bacterial infection (OR = 1.956, 95% CI = 1.026–3.730, *p* = 0.042).

The mean length of stay was 32.9 ± 38.0 for patients with COPD and 17.7 ± 17.5 days for patients without COPD, with *p* < 0.001. The results are summarized in Table 2.

**TABLE 2:** Complications from COVID‐19 for patients with or without COPD.

Complications	Univariate analysis	*p*	Multivariate analysis^a^	*p*
	Odds ratios and 95% CI		Odds ratios and 95% CI	
Respiratory failure^b^	3.369 (2.476–5.349)	< 0.001	2.625 (1.560–4.417)	< 0.001
Invasive mechanical ventilation^b^	3.485 (2.275–5.339)	< 0.001	2.196 (1.186–4.069)	0.012
Invasive mechanical ventilation > 95 h^b^	3.156 (1.553–6.415)	0.001	4.374 (1.485–12.881)	0.007
Require systemic steroid treatment^b^	2.994 (2.075–4.320)	< 0.001	2.565 (1.550–4.244)	< 0.001
Intensive care unit admission^b^	3.478 (2.361–5.125)	< 0.001	1.842 (1.054–3.218)	0.032
COVID pneumonia^b^	4.456 (3.163–6.532)	< 0.001	3.054 (1.787–5.219)	< 0.001
ARDS	1.523 (0.834–2.780)	0.171		
Shock	3.053 (1.933–4.821)	< 0.001	1.619 (0.837–3.132)	0.153
Acute kidney injury	1.653 (0.577–4.737)	0.350		
Secondary bacterial infection^b^	3.729 (2.374–5.858)	< 0.001	1.956 (1.026–3.730)	0.042
Secondary viral infection	1.761 (0.612–5.067)	0.294		
Inpatient mortality	3.941 (2.458–6.318)	< 0.001	1.302 (0.657–2.583)	0.450
30‐day mortality	2.910 (1.621–5.223)	< 0.001	1.225 (0.568–2.643)	0.604

^a^ Adjustment done for confounders including Charlson Comorbidity Index, serum albumin level, and lymphocyte count.

^b^ Factors that are statistically significant after adjustment for confounders.

### 3.3 Disease Severity and Complications for Patients With COPD of Different Blood Eosinophil Count on Admission and Patients Without COPD

To assess the impact of blood eosinophil count on admission on severity of COVID‐19, patients with COPD were divided into two groups based on blood eosinophil count on admission. Patients with admission blood eosinophil count ≥ 150 cells/μL and patients with COPD and admission blood eosinophil count < 150 cells/μL were compared with those without COPD. Patients without COPD served as the control group for comparison. At univariate logistic regression, patients with COPD with admission blood eosinophil count ≥ 150 cells/μL and < 150 cells/μL were significantly more likely to develop respiratory failure; require invasive mechanical ventilation; require invasive mechanical ventilation of more than 95 h, intensive care unit admission, and systemic corticosteroid treatment; develop shock and secondary bacterial infection; and have inpatient mortality and 30‐day mortality.

In multivariate logistic regression, only patients with COPD and admission blood eosinophil count ≥ 150 cells/μL were found have severe diseases, but not those with admission blood eosinophil count < 150 cells/μL. Patients with COPD and admission blood eosinophil count ≥ 150 cells/μL were more likely to develop respiratory failure (OR = 5.235, 95% CI = 2.088–13.122, *p* < 0.001); require invasive mechanical ventilation (OR = 2.433, 95% CI = 1.022–5.791, *p* = 0.045), invasive mechanical ventilation of more than 95 h (OR = 7.257, 95% CI = 2.076–25.369, *p* = 0.002), and intensive care unit admission (OR = 2.214, 95% CI = 1.004–4.881, *p* = 0.049), and systemic corticosteroid treatment (OR = 4.694, 95% CI = 2.036–10.820, *p* < 0.001); and develop secondary bacterial infection (OR = 3.692, 95% CI = 1.084–12.572, *p* = 0.037).

The mean length of stay was 39.2 ± 49.8 days for patients with COPD and admission blood eosinophil count ≥ 150 cells/μL, 30.2 ± 31.5 days for patients with COPD and admission blood eosinophil count < 150 cells/μL, and 17.7 ± 17.5 days for patients without COPD, with *p* < 0.001. The results are summarized in Table 3.

**TABLE 3:** Complications from COVID‐19 for patients with or without COPD and of different eosinophil count on admission.

Complications	Blood eosinophil count on admission for COPD patients	Univariate analysis Odds ratios and 95% CI	*p*	Multivariate analysis^a^	*p*
Respiratory failure	< 150 cells/μL	3.171 (2.032–4.947)	< 0.001	1.798 (0.961–3.365)	0.066
	≥ 150 cells/μL^b^	5.200 (2.461–10.989)	< 0.001	5.235 (2.088–13.122)	< 0.001
Invasive mechanical ventilation	< 150 cells/μL	3.470 (2.107–5.716)	< 0.001	2.041 (0.946–4.401)	0.069
	≥ 150 cells/μL^b^	3.520 (1.689–5.716)	< 0.001	2.433 (1.022–5.791)	0.045
Invasive mechanical ventilation > 95 h	< 150 cells/μL	2.677 (1.112–6.446)	0.028	2.396 (0.488–11.754)	0.282
	≥ 150 cells/μL^b^	4.314 (0.473–12.633)	0.008	7.257 (2.076–25.369)	0.002
Require systemic steroid treatment	< 150 cells/μL	2.559 (1.671–3.919)	< 0.001	1.795 (0.970–3.322)	0.063
	≥ 150 cells/μL^b^	4.457 (2.212–8.981)	< 0.001	4.694 (2.036–10.820)	< 0.001
Intensive care unit admission	< 150 cells/μL	3.518 (2.236–5.536)	< 0.001	1.609 (0.792–3.268)	0.188
	≥ 150 cells/μL^b^	3.388 (1.723–6.662)	< 0.001	2.214 (1.004–4.881)	0.049
COVID pneumonia	< 150 cells/μL^b^	3.982 (2.596–6.109)	< 0.001	2.719 (1.407–8.256)	0.003
	≥ 150 cells/μL^b^	6.173 (3.270–11.652)	< 0.001	3.686 (1.606–8.460)	0.002
ARDS	< 150 cells/μL	1.694 (0.856–3.350)	0.130		
	≥ 150 cells/μL	1.140 (0.346–3.751)	0.829		
Shock	< 150 cells/μL	2.877 (1.671–4.951)	< 0.001	1.335 (0.571–3.121)	0.505
	≥ 150 cells/μL	3.480 (1.624–7.457)	< 0.001	2.088 (0.846–5.156)	0.110
Acute kidney injury	< 150 cells/μL	0.830 (0.277–4.958)	0.830		
	≥ 150 cells/μL	2.805 (0.650–12.117)	0.167		
Secondary bacterial infection	< 150 cells/μL	2.997 (1.812–4.958)	< 0.001	1.430 (0.672–3.041)	0.353
	≥ 150 cells/μL^b^	7.390 (2.620–20.850)	< 0.001	3.692 (1.084–12.572)	0.037
Secondary viral infection	< 150 cells/μL	1.939 (0.590–6.371)	0.275		
	≥ 150 cells/μL	3.127 (0.732–13.353)	0.124		
Inpatient mortality	< 150 cells/μL	3.642 (2.076–6.389)	< 0.001	1.057 (0.435–2.565)	0.903
	≥ 150 cells/μL	4.665 (2.1647–10.053)	< 0.001	1.709 (0.668–4.368)	0.263
30‐day mortality	< 150 cells/μL	2.453 (1.188–5.066)	0.015	0.822 (0.291–2.323)	0.711
	≥ 150 cells/μL	4.040 (1.646–9.918)	0.002	1.990 (0.731–5.419)	0.528

^a^ Adjustment done for confounders including Charlson Comorbidity Index, serum albumin level and lymphocyte count.

^b^ Factors that are statistically significant after adjustment for confounders.

### 3.4 Subgroup Analysis

Subgroup analysis was performed among patients with Group A/B COPD and also Group E COPD, stratified into two groups by admission blood eosinophil count, and compared with patients without COPD, as the control group.

Among patient with Group A/B COPD, 28 had admission blood eosinophil count ≥ 150 cells/μL, and 70 had admission blood eosinophil count < 150 cells/μL. Patients with admission blood eosinophil count ≥ 150 cells/μL had significantly increased risks for developing respiratory failure, requiring IMV (including > 95 h), requiring systemic corticosteroid, requiring ICU admission, and developing COVID‐19 pneumonia, which was consistent with results from the main cohort. The results are summarized in Table S1.

Among patients with Group E COPD, 12 had admission blood eosinophil count ≥ 150 cells/μL, and 23 had admission blood eosinophil count < 150 cells/μL. Due to small sample size in this COPD subgroup, there was no significant results from multivariate analysis. The results are summarized in Table S2.

## 4 Discussion

Our study suggested that the blood eosinophil count on admission could have significant prognostic implications among elderly patients with COPD. Elderly patients with COPD and admission blood eosinophil count ≥ 150 cells/μL were found to have significantly increased risks of severe COVID‐19 with the development of develop respiratory failure, requiring invasive mechanical ventilation, requiring intensive care unit, requiring systemic corticosteroid treatment, and developing secondary bacterial infection. This group of patients also have the longest length of stay upon admission for COVID‐19. The findings of this study not only concur with previous reports that COPD being a risk factor of severe COVID‐19 but also identify a subgroup with admission blood eosinophil count ≥ 150 cells/μL who have the worse prognosis with more severe COVID‐19.

COPD is one of the most common comorbidities among elderly patients. Both advanced age and underlying comorbidities are well reported to be poor prognostic factors in COVID‐19. To identify a higher risk subgroup within this population would be important for risk stratification. This would be important when various therapeutic agents, including antiviral and adjunctive therapies are developed for COVID‐19. Elderly patients with COPD and admission blood eosinophil count ≥ 150 cells/μL but not those with admission blood eosinophil count < 150 cells/μL were found to be associated with severe COVID‐19 disease with respiratory and systemic complications, in multivariate analysis adjusted for CCI, serum albumin level, and lymphocyte count. They also required longer length of stay compared with those with COPD and admission blood eosinophil count < 150 cells/μL and patients without COPD. Our finding echoes on the previous reports on the potential role of admission blood eosinophil count among COPD patients.

COPD phenotyping using serum eosinophil level was extensively studied in recent years. Most of the studies are done using blood eosinophil count at stable state to phenotype the patients and assess the subsequent outcomes. Patients with eosinophilic phenotype based on baseline eosinophil level were found to have increased risks of risk of future exacerbations and is associated with improved response to treatment with inhaled corticosteroids [36]. On the other hand, Pavord et al. reported that using 2% baseline eosinophil count as a threshold at clinical stable state, patients with COPD with lower blood eosinophil counts had more pneumonia events than those with higher counts [37]. Study suggested that there was significant positive correlation of eosinophil counts between stable COPD and at COPD exacerbation [38]. As such, using the admission blood eosinophil count can serve as a good surrogate for the phenotype of COPD. COPD phenotyping by type of inflammation based on blood eosinophil level affect not only patients' prognosis but also the treatment of choice, including inhaled corticosteroid treatment [39].

The role of the eosinophil level at the time of hospitalization for COPD exacerbation remains controversial and conflicting results from different studies [40, 41, 42]. Zhang et al. reported that increased eosinophils measured at index hospitalization with the risk of all‐cause death among patients hospitalized for COPD exacerbation [12]. Couillard et al. reported that eosinophilia was associated with an increased risk of 12‐month COPD‐related readmission, an increased risk of 12‐month all‐cause readmission, and a shorter time to first COPD‐related readmission [41]. Greater blood eosinophil cell counts during a first hospitalization for COPD were also reported to be able to predict increased susceptibility to up to two readmissions [42]. In Hasegawa et al. that included 3084 patients hospitalized for acute exacerbation of COPD, blood eosinophil count at the time of COPD exacerbation did not affect the in‐hospital course, including the length of stay and in‐hospital mortality [43]. Wu et al. reported that patients with eosinophil count ≥ 2% had the features of a shorter length of hospital stay and lower doses of systemic steroids but more frequent readmissions [44]. Csoma et al. reported that there was no increased risk of earlier recurring moderate or severe relapses in patients hospitalized with eosinophilic exacerbations of COPD [40].

The discrepancy in the results from these studies could be due to the different definitions of eosinophilia used in these studies. Some studies used absolute eosinophil count, whereas some used blood eosinophil percentage, which could be affected by the neutrophil and lymphocyte count that constituted the white blood cell count, that is, the denominator. At the same time, the outcomes of interest were also different across these studies. Studying the role of blood eosinophil level at time of COPD exacerbation and the immediate/short‐term outcome in index admission and the medium‐ to long‐term outcome may lead to completely different findings, as these outcomes occur at different time point. Immediate/short‐term outcomes such as length of stay and mortality in index admission might be related to the severity of the exacerbation, whereas medium‐ to long‐term outcome might be affected by the severity of underlying COPD instead. These could explain the conflicting results among these studies. Nonetheless, blood eosinophil level remains the most important and readily available biomarkers in COPD in both prognostication and personalized pharmacotherapy. The role of blood eosinophil level and prognosis are still worth studying, especially in different clinical scenario, such as COVID‐19 in our study.

Our study findings provided a readily available tool to help to stratify the risks among elderly patients of COPD infected by COVID‐19. Elderly and COPD have been considered to be one of the independent prognostic factors in COVID‐19. But from our study findings, the phenotype of COPD also plays an important role in prognostication. The blood eosinophil level, at the cutoff of 150 cells/μL, can help to predict the development of respiratory and systemic complications among patients with COPD infected by COVID‐19. For COPD and admission blood eosinophil count ≥ 150 cells/μL, as they are more prone to develop severe COVID‐19 infection, they should be considered to have more aggressive treatment for COVID‐19 earlier in the disease course to prevent the development of complications. Early aggressive treatment for these patients may not only be able to prevent major complications from COVID‐19 and shorten their hospital length of stay, but it may also have long term benefits for these patients. Nowadays, apart from single antiviral, combination antiviral [28, 29, 30] and the use with adjunctive therapies have been studied in various clinical settings with benefits demonstrated [21, 22, 24, 25, 26, 27]. By preventing developing respiratory complications, this may help to prevent any potential lung damage including post‐inflammatory fibrosis in these patients, who already have compromised lung function due to underlying COPD. Furthermore, whether inhaled corticosteroid can help to reduce the risks of complications among these patients would be worth investigating. Although inhaled corticosteroid should only be prescribed for patients with COPD with history of exacerbation and of eosinophilic phenotype, the finding of our study, as well as that has been reported in this literature [19, 45, 46], may suggest for further research direction on this area. Inhaled corticosteroids shall be continued among COPD patients who are indicated for the treatment. For those with eosinophilic phenotype or high admission blood eosinophil count, whether add‐on short course ICS may provide extra benefits would be worth investigating. Furthermore, with the development and advances of various COVID‐19 specific therapy, identification of specific high‐risk subgroup based on admission blood eosinophil count would allow personalized treatment for elderly COPD patients based their risk profile [47, 48, 49, 50]. The role of COVID‐19 vaccination needs not be overemphasized among elderly COPD patients, in particular those with eosinophilic phenotype.

One of the possible factors to affect the blood eosinophil levels of the patients is the use of ICS. Kreindler et al. reported that among steroid‐naïve patients with COPD, the median (IQR) changes in eosinophil count for ICS‐treated patients were −30 (−90–10) cells/μL at week 6 and −30 (−90–20) cells/μL at Week 12 [51]. The authors concluded that ICS‐containing treatment had a small effect on peripheral blood eosinophils in steroid‐naïve patients with COPD. Mathioudakis et al. also suggested that the change in blood eosinophil count after ICS administration may predict clinical response to ICS therapy in patients with moderate to severe COPD at risk of exacerbations [52]. In our cohort, there were numerically more patients on ICS in the high eosinophil group with admission blood eosinophil count ≥ 150 cells/μL but the difference was not statistically significant with *p* value of 0.527. The use of ICS might not have major implications on the study outcomes given a nonsignificant difference in proportion of COPD on ICS in the two groups.

One of the strengths of our study lies on the healthcare policy in Hong Kong. All patients with COVID‐19 regardless of disease severity need to be hospitalized in public hospital or community treatment facility, which are managed by the Hospital Authority under isolation order from the government, according to the public health policy. For patients with moderate to severe disease, they will be managed in the isolation ward of acute hospital, whereas mild and asymptomatic cases are managed in Hong Kong Infection Control Centre. Hong Kong government did not adopt home‐based management. The discharge criteria in Hong Kong are having repeated respiratory specimens being negative for SAR‐CoV2. These public health policies allow us to have a more comprehensive assessment and comparison by including all patients with confirmed COVID‐19 in Hong Kong within the captioned period, from asymptomatic diseases to fatal cases.

The limitation in our study includes lack of data of viral load for the patients. But the need for treatment for COVID‐19 was potential surrogates, as asymptomatic patients with low viral load would not need treatment.

## 5 Conclusions and Implications

Patients with COPD and admission blood eosinophil count ≥ 150 cells/μL, but not those with admission blood eosinophil count < 150 cells/μL, have significantly increased risks of developing respiratory and systemic complications from COVID‐19 infection, when compared with patients without COPD.

## Author Contributions

W.C.K. and Y.F.S. were involved with study concept and design, analysis and interpretation of data, acquisition of data, drafting of manuscript, and approval of the final version of the manuscript. A.R.T., T.C.C.T., J.C.M.H., D.C.L.L., and M.S.M.I. were involved with critical revision of the manuscript for important intellectual content and approval of the final version of the manuscript. I.F.N.H. was involved with the study concept and design, analysis and interpretation of data, drafting of manuscript, critical revision of the manuscript for important intellectual content, study supervision, and approval of the final version of the manuscript.

## Ethics Statement

The study was approved by the Institutional Review Board of the University of Hong Kong and Hospital Authority Hong Kong West Cluster (UW 22‐089), and all methods were performed in accordance with the relevant guidelines and regulations provided by the IRB. Patient consent was waived by the IRB as it is a retrospective study without active patient recruitment, but the data were already de‐identified. The study was conducted in compliance with the Declaration of Helsinki. Patient data were maintained with confidentiality throughout the study.

## Conflicts of Interest

The authors declare no conflicts of interest.

## Supporting information


**Table S1:** Complications from COVID‐19 for patients with or without COPD and of different eosinophil count on admission among subgroup with Group A/B COPD compared with patients without COPD.
**Table S2**: Complications from COVID‐19 for patients with or without COPD and of different eosinophil count on admission among subgroup with Group E COPD compared with patients without COPD.

## Data Availability

All available data are presented in the manuscript and no additional data will be provided. Our data is not available for sharing.
