# Supplementary material for: Implication of Admission Eosinophil Count and Prognosis of Coronavirus Disease 2019 (COVID‐19) in Elderly Patients With COPD: A Territory‐Wide Cohort Study
Source: Clin Respir J. 2025 Mar 27;19(4):e70070. doi: 10.1111/crj.70070 (PMC11947431; doi:10.1111/crj.70070)
Supplement: Supplementary file 1 — Table S1: Complications from COVID‐19 for patients with or without COPD and of different eosinophil count on admission among subgroup with Group A/B COPD compared with patients without COPD. Table S2: Complications from COVID‐19 for patients with or without COPD and of different eosinophil count on admission among subgroup with Group E COPD compared with patients without COPD. [file CRJ-19-e70070-s001.docx]

**Supplementary Table 1: Complications from COVID-19 for patients with or without COPD and of different eosinophil count on admission among subgroup with Group A/B COPD compared with patients without COPD**

| **Complications** | **Blood eosinophil count on admission for COPD patients** | **Univariate analysis**  **Odds ratios and 95% CI** | **p value** | **Multivariate analysis**^†^ | **p value** |
| --- | --- | --- | --- | --- | --- |
| Respiratory failure | < 150 cells/µL | 3.299 (1.974 – 5.512) | <0.001 | 1.828 (0.868 – 3.852) | 0.113 |
|  | > 150 cells/µL* | 4.536 (1.918 – 10.725) | <0.001 | 3.286 (1.250 – 8.638) | 0.016 |
| Invasive mechanical ventilation | < 150 cells/µL | 5.905 (2.679 – 13.018) | <0.001 | 1.796 (0.691 – 4.667) | 0.229 |
|  | > 150 cells/µL * | 3.680 (2.100 – 6.447) | <0.001 | 3.741 (1.380 – 10.143) | 0.010 |
| Invasive mechanical ventilation > 95 hours | < 150 cells/µL | 3.722 (1.531 – 9.052) | 0.004 | 3.795 (0.750 – 19.211) | 0.107 |
|  | > 150 cells/µL* | 6.617 (2.203 – 19.879) | <0.001 | 10.978 (2.733 – 44.091) | <0.001 |
| Require systemic steroid treatment | < 150 cells/µL* | 2.859 (1.744 – 4.686) | <0.001 | 2.397 (1.142 – 5.031) | 0.021 |
|  | > 150 cells/µL* | 3.566 (1.604 – 7.297) | 0.002 | 2.864 (1.170 – 7.011) | 0.021 |
| Intensive care unit admission | < 150 cells/µL | 3.689 (2.207 – 6.160) | <0.001 | 1.327 (0.556 – 3.165) | 0.524 |
|  | > 150 cells/µL* | 6.125 (2.876 – 13.044) | <0.001 | 3.158 (1.296 – 7.696_ | 0.011 |
| COVID pneumonia* | < 150 cells/µL* | 4.253 (2.615 – 6.918) | <0.001 | 2.572 (1.192 – 5.548) | 0.016 |
|  | > 150 cells/µL* | 7.806 (3.619 – 16.835) | <0.001 | 3.293 (1.289 – 8.409) | 0.013 |
| ARDS | < 150 cells/µL | 2.073 (1.005 – 4.277) | 0.048 |  |  |
|  | > 150 cells/µL | 1.686 (0.502 – 5.665) | 0.398 |  |  |
| Shock | < 150 cells/µL | 2.995 (1.626 – 5.516) | <0.001 | 1.418 (0.531 – 3.786) | 0.486 |
|  | > 150 cells/µL* | 5.674 (2.519 – 12.779) | <0.001 | 2.662 (1.006 – 7.045) | 0.049 |
| Acute kidney injury | < 150 cells/µL | 1.567 (0.368 – 6.664) | 0.543 |  |  |
|  | > 150 cells/µL | 4.098 (0.934 – 17.982) | 0.062 |  |  |
| Secondary bacterial infection | < 150 cells/µL | 3.596 (1.955 – 6.615) | <0.001 | 1.737 (0.677 – 4.456) | 0.251 |
|  | > 150 cells/µL | 6.834 (2.056 – 22.717) | 0.002 | 2.475 (0.695 – 8.815) | 0.162 |
| Secondary viral infection | < 150 cells/µL | 0.823 (0.111 – 6.115) | 0.849 | 0 (0-0) | 0.998 |
|  | > 150 cells/µL | 4.367 (0.993 – 19.212) | 0.051 | 1.920 (0.214 – 17.239) | 0.560 |
| In-patient mortality | < 150 cells/µL | 3.278 (1.976 – 7.032) | <0.001 | 0.808 (0.273 – 2.390) | 0.700 |
|  | > 150 cells/µL | 6.423 (2.764 – 14.927) | <0.001 | 1.229 (0.429 – 3.521) | 0.701 |
| 30-day mortality | < 150 cells/µL | 2.952 (1.365 – 6.387) | 0.006 | 0.869 (0.270 – 2.799) | 0.814 |
|  | > 150 cells/µL | 4.974 (1.840 – 13.445) | 0.002 | 1.369 (0.441 – 4.253) | 0.587 |

*Factors that are statistically significant after adjustment for confounders

^†^Adjustment done for confounders including Charlson Comorbidity Index, serum albumin level and lymphocyte count

**Supplementary Table 2: Complications from COVID-19 for patients with or without COPD and of different eosinophil count on admission among subgroup with Group E COPD compared with patients without COPD**

| **Complications** | **Blood eosinophil count on admission for COPD patients** | **Univariate analysis**  **Odds ratios and 95% CI** | **p value** | **Multivariate analysis**^†^ | **p value** |
| --- | --- | --- | --- | --- | --- |
| Respiratory failure | < 150 cells/µL | 2.835 (1.196 – 6.721) | 0.018 | 1.649 (0.546 – 4.982) | 0.375 |
|  | > 150 cells/µL | 7.560 (1.652 – 34.603) | 0.009 | - | 0.999 |
| Invasive mechanical ventilation | < 150 cells/µL | 2.953 (1.081 – 8.062) | 0.035 | 2.679 (0.716 – 10.026) | 0.143 |
|  | > 150 cells/µL | 0 (0-0) | 0.999 | 0 (0-0) | 0.999 |
| Invasive mechanical ventilation > 95 hours | < 150 cells/µL | 0 (0-0) | 0.998 |  |  |
|  | > 150 cells/µL | 0 (0-0) | 0.999 |  |  |
| Require systemic steroid treatment | < 150 cells/µL | 1.843 (0.809 – 4.200) | 0.146 | 0.874 (0.282 – 2.707) | 0.815 |
|  | > 150 cells/µL | 8.446 (1.845 – 38.663) | 0.006 | - | 0.999 |
| Intensive care unit admission | < 150 cells/µL | 3.092 (1.258 – 7.599) | 0.014 | 2.196 (0.658 – 7.331) | 0.201 |
|  | > 150 cells/µL | 0 (0-0) | 0.999 | 0 (0-0) | 0.999 |
| COVID pneumonia | < 150 cells/µL |  |  |  |  |
|  | > 150 cells/µL |  |  |  |  |
| ARDS | < 150 cells/µL | 0.639 (0.085 – 4.779) | 0.662 |  |  |
|  | > 150 cells/µL | 0 (0-0) | 0.999 |  |  |
| Shock | < 150 cells/µL | 2.522 (0.846 – 7.516) | 0.097 |  |  |
|  | > 150 cells/µL | 0 (0-0) | 0.999 |  |  |
| Acute kidney injury | < 150 cells/µL | 0 (0-0) | 0.998 |  |  |
|  | > 150 cells/µL | 0 (0-0) | 0.999 |  |  |
| Secondary bacterial infection* | < 150 cells/µL | 1.875 (0.767 – 4.579) | 0.168 | 1.032 (0.295 – 3.612) | 0.960 |
|  | > 150 cells/µL | 9.021 (1.162 – 70.023) | 0.035 | - | 0.999 |
| Secondary viral infection | < 150 cells/µL | 2.581 (0.337 – 19.572) | 0.361 |  |  |
|  | > 150 cells/µL | 0 (0-0) | 0.999 |  |  |
| In-patient mortality | < 150 cells/µL | 3.380 (1.130 – 10.116_ | 0.029 | 1.574 (0.389 – 6.371) | 0.525 |
|  | > 150 cells/µL | 1.460 (0.187 – 11.414) |  | 0.791 (0.081 – 7.719) | 0.840 |
| 30-day mortality | < 150 cells/µL | 1.040 (0.138 – 7.819) | 0.970 |  |  |
|  | > 150 cells/µL | 2.080 (0.265 – 16.322) | 0.486 |  |  |

*Factors that are statistically significant after adjustment for confounders

^†^Adjustment done for confounders including Charlson Comorbidity Index, serum albumin level and lymphocyte count
